# Rekonstruktive Beckenbodenchirurgie – Update 2024: prolapsassoziierte Symptome und deren Heilung

**DOI:** 10.1007/s00120-023-02247-6

**Published:** 2023-12-28

**Authors:** B. Liedl, M. Barba, M. Wenk

**Affiliations:** 1 Zentrum für Rekonstruktive Urogenitalchirurgie, Urologische Klinik München-Planegg, Germeringer Str. 32, 82152 München-Planegg, Deutschland; 2 Abteilung für Urologie, Kreiskrankenhaus Ebersberg, akad. Lehrkrankenhaus der technischen Universität München, Klinikum rechts der Isar, München, Deutschland; 3 Klinik für Urologie und Urochirurgie, Universitätsmedizin Mannheim, Universität Heidelberg, Mannheim, Deutschland

**Keywords:** Urogenitaler Deszensus, Beckenschmerz, Überaktive Blase, Obstruktive Miktion, Stuhlinkontinenz, Pelvic organ prolapse, Pelvic pain, Overactive bladder, Obstructive Micturition, Fecal incontinence

## Abstract

Der urogenitale Deszensus und die assoziierte Symptomatik von Harninkontinenz, Stuhlinkontinenz, Blasen- und Stuhlentleerungsstörung und Schmerzen treten häufig auf und sind Volkskrankheit mit starker Beeinträchtigung der Lebensqualität und Verursachung großer Kosten für das Gesundheitssystem. Neuere Erkenntnisse der funktionellen Anatomie und Pathophysiologie dieser Beckenbodendysfunktionen zeigen, wie bindegewebige Lockerungen bzw. Defekte zu diesen Dysfunktionen führen. Es werden Ergebnisse der PROpel-Studie (ClinicalTrials.gov-Identifier: NCT00638235) gezeigt, in der eine ausführliche Symptombeobachtung mit PROM („patient-related outcome measures“) präoperativ und postoperativ erfolgte. Eine ligamentäre vaginale Prolapskorrektur erlaubt mittlerweile Symptomheilungen in hohen Prozentsätzen, sowohl von Harndranginkontinenz (bis zu 82 %), Nykturie (bis zu 92 %), Blasenentleerungsstörung (bis zu 87 %), Stuhlinkontinenz (58–72 %), obstruktiver Defäkation (71–84 %) und Schmerzen (53–90 %), wenn sie durch vaginalen Prolaps verursacht sind. Symptome treten bei Frauen mit POP-Q-Stadium II (Pelvic Organ Prolapse-Quantification) ähnlich häufig auf wie bei Frauen mit POP-Q-Stadium III–IV, und die anatomisch gute operative Korrektur führt zu ähnlich hohen Heilungsraten. Bei anatomisch guter (Responder) im Vergleich zur weniger guten Prolapskorrektur (Non-Responder) wurden signifikante Unterschiede der Heilungsraten sowohl für Blasenentleerungsstörung als auch für Harndranginkontinenz und Nykturie gesehen. Die künftige Beckenbodenchirurgie sollte als Ziel die Symptomheilung haben und zur Verbesserung der Lebensqualität führen. Die derzeit verwendeten unterschiedlichen Techniken zur Prolapskorrektur müssen diesbezüglich auf den Prüfstand gestellt werden.

## Abstract (en)

Pelvic organ prolapse (POP) and associated symptoms of urinary incontinence, fecal incontinence, obstructive micturition, defecation, and pain are frequent and a widespread disease with relevant reduction of quality of life and high costs. New insights into functional anatomy and pathophysiology of these pelvic floor dysfunctions let us recognize how ligamentous laxities/defects lead to these dysfunctions. Results of the PROpel study (ClinicalTrials.gov-Identifier: NCT00638235) are shown in which a detailed observation of symptoms (patient-related outcome measures) pre- and postoperatively was performed. Ligamentous vaginal repair of POP enables symptom cure in high percentages for urinary urge incontinence (up to 82%), nocturia (up to 92%), obstructive micturition (up to 87%), fecal incontinence (58–72%), obstructive defecation (71–84%), and pain (53–90%), if caused by POP. Women with POP‑Q stage 2 have similar symptom frequencies as women with POP‑Q stage 3–4, and also similar cure rates of their symptoms. If good anatomical prolapse repair (in responders) was achieved, the cure rates for obstructive micturition, urinary urgency incontinence, and nocturia were significantly higher than in those women with less effective surgical repair. In the future, pelvic floor surgery should have symptom cure as the primary objective and should lead to improved quality of life. The different, currently performed techniques for POP repair have to be investigated concerning this matter.

## Hintergrund

Die Prävalenz des urogenitalen Deszensus bei Frauen variiert zwischen 1 und 31 %, wenn die Diagnose durch Symptomerfassung erfolgt und zwischen 10–50 %, wenn sie durch eine vaginale Untersuchung gestellt wird [1]. Die Prävalenz symptomatischer Beckenbodendysfunktionen steigt mit zunehmendem Alter, Parität und Körpergewicht an [2]. Auch die Häufigkeit der Nykturie bei Frauen steigt mit zunehmenden Alter auf ca. 10–30 % (50-Jährige) und 30–50 % (70-Jährige) ebenso wie die Häufigkeit der Harninkontinenz [3]. Beim urogenitalen Deszensus mit den entsprechenden Symptomen handelt es sich somit um eine Volkskrankheit, die die Lebensqualität stark beeinträchtigt und große Kosten verursacht [4].

Beim urogenitalen Deszensus handelt es sich um eine Volkskrankheit

In 1990 und 1993 haben Petros und Ulmsten [5, 6] die Pathophysiologie der prolapsbedingten Belastungsharninkontinenz, der Harndrangbeschwerden sowie der Blasenentleerungsstörungen beschrieben, 1996 die Verursachung von Beckenschmerzen durch einen apikalen Prolaps [7] und später die Mechanismen der Entstehung prolapsbedingter obstruktiver Defäkation und Stuhlinkontinenz postuliert [8]. Es wurden Techniken zur ligamentären Prolapskorrektur entwickelt und erste Ergebnisse von Symptomheilungen berichtet [9]. Im Folgenden sollen die neuen pathophysiologischen Mechanismen der Symptomentstehung und mögliche chirurgische Heilungsraten dargestellt werden.

## Funktionelle Anatomie des Beckenbodens

**Abb. 1:**
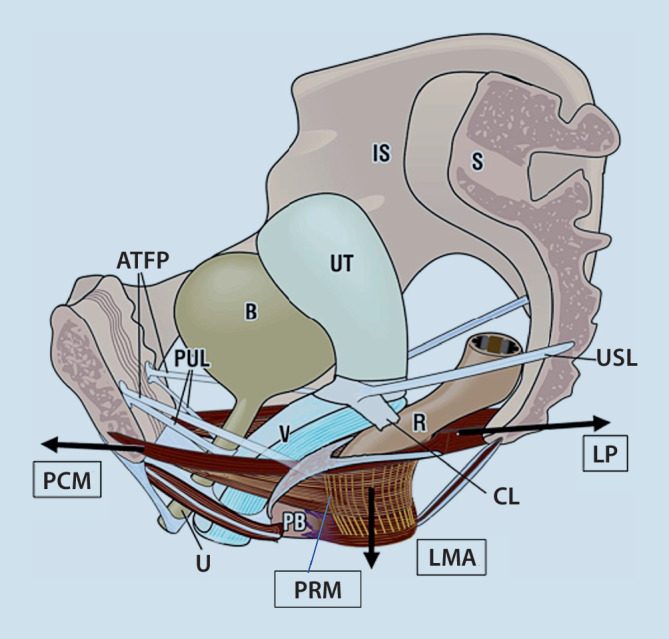
Wichtige Ligamente des Beckenbodens: *PUL* pubourethrales Ligament, *ATFP* Arcus tendineus fasciae pelvis, *CL* kardinales Ligament, *USL* uterosakrales Ligament, *PB* Perinealkörper. Wichtige Muskeln des Beckenbodens: *PCM* M. pubococcygeus, *LMA* longitudinaler Muskel des Anus, *LP* Levatorplatte, *PRM* M. puborectalis, *U* Urethra, *B* Blase, *Ut* Uterus, *V* Vagina, *R* Rektum, *PB* Perinealkörper. Die *schwarzen Pfeile* geben die Zugrichtungen an. (Mod. nach Petros [9])

Um die Dysfunktionen des Beckenbodens und auch die geeignete Prolapschirurgie zu erlernen, ist es zunächst wichtig, den bindegewebigen Stützapparat des kleinen Beckens mit den wichtigsten Faszien und Bändern dreidimensional zu verstehen (Abb. 1). Zu nennen sind das pubourethrale Ligament (retropubische Insertion), das sakrouterine Ligament (Fixation dorsal in Höhe S2–S4), das kardinale Ligament (seitliche Fixation ventral der Spina ischiadica), der Arcus tendineus fasciae pelvis und der Perinealkörper am Perineum. Zusammen mit der pubozervikalen Faszie, der rektovaginalen Faszie, dem extraurethralen Ligament und der suburethralen Hängematte (Hammock) halten sie die Organe des kleinen Beckens – insbesondere die Harnblase, Harnröhre, Uterus, Anorektum – in der optimalen Lage, so dass die Binnenmuskeln des kleinen Beckens optimal funktionieren können [9].

Für den Verschluss der Harnröhre und des Anorektums – insbesondere bei Belastung – sind Aktionen mehrerer Muskeln beteiligt (Abb. 1; Tab. 1). Zur Erlangung einer Belastungsharnkontinenz ist nicht nur der Rhabdosphinkter wichtig, sondern v. a. der M. pubococcygeus (PCM) und der M. puborectalis (PRM), die die vordere Scheidenwand nach ventral ziehen. Bei intaktem Lig. pubourethrale ziehen die Levatorplatte (LP) und der longitudinale Muskel des Anus (LMA) nach dorsal und kaudal, so dass eine Streckung und Abknickung der proximalen Urethra resultiert. Auch bei einer normalen Miktion sind aktive Muskelaktionen beteiligt, um die proximale Urethra aktiv zu öffnen. Dies geschieht durch die Kontraktion von LP und LMA bei zeitgleicher Erschlaffung des Rhabdosphinkters, des PCM und des PRM [10, 11]. Für die Stuhlkontinenz und die. Stuhlentleerung sind ebenfalls Muskelaktionen erforderlich (Tab. 1; [8]).

**Tab. 1:** Muskelaktionen zum Verschluss und zur Öffnung des Blasenauslasses und des Anorektums

	Muskelkontraktion	Muskelerschlaffung
Belastungsharnkontinenz	PCM, PRM, LP, LMA, Rhabdosphinkter	Detrusormuskel
Miktion	LP, LMA, Detrusor	PCM, PRM, Rhabdosphinkter
Stuhlkontinenz	PRM, LP, LMA, Analsphinkter	–
Stuhlentleerung	LP, LMA	PRM, Analsphinkter

*PCM* M. pubococcygeus, *LMA* longitudinaler Muskel des Anus, *LP* Levatorplatte, *PRM* M. puborectalis

## Pathophysiologie der Beckenbodendysfunktionen

Wesentlich für die Entwicklung eines vaginalen Deszensus sind bindegewebige Lockerungen bzw. Defekte, die sich mit zunehmendem Alter, nach vaginalen Geburten und auch durch angeborene Defekte einstellen [6, 12, 13].

Wesentlich für die Entwicklung eines vaginalen Deszensus sind bindegewebige Lockerungen bzw. Defekte

**Abb. 2:**
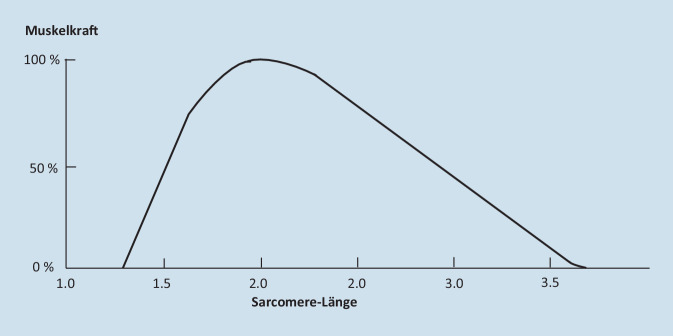
Abhängigkeit der Muskelkraft von der Muskel (Sarkomer)-Länge. [24]

Durch den Descensus urogenitalis werden auch Band- und Muskelansatzpunkte/-areale an der Scheide verändert, was zur Verkürzung oder Verlängerung der Muskelfasern führt. Da die genannten Beckenbodenmuskeln alle quergestreift sind und aus Sarkomeren bestehen, unterliegen sie der Abhängigkeit der Muskelkraft von der Muskellänge, wie es Gordon et al. 1966 [24] beschrieben haben (Abb. 2). Eine Verlängerung oder Verkürzung einer Muskelfaser führt danach unmittelbar zu einem raschen Verlust der Muskelkraft bis hin zur 0 %, da der Muskel seine Kraft nur in einem bestimmten Optimum der Sarkomerlänge entfalten kann. Konsekutiv kommt es zu Funktionsverlusten der in Tab. 1 genannten Funktionen. Bei Behebung der Muskelverlängerung bzw. der -verkürzung – durch Behebung des Deszensus – kommt es zur unmittelbaren Wiedererlangung der Muskelkraft und der Funktion. Dies trifft sowohl für die Verschlusskräfte (Belastungsharninkontinenz, Stuhlkontinenz) als auch die Öffnungskräfte (Miktion, Stuhlentleerung) zu.

**Abb. 3:**
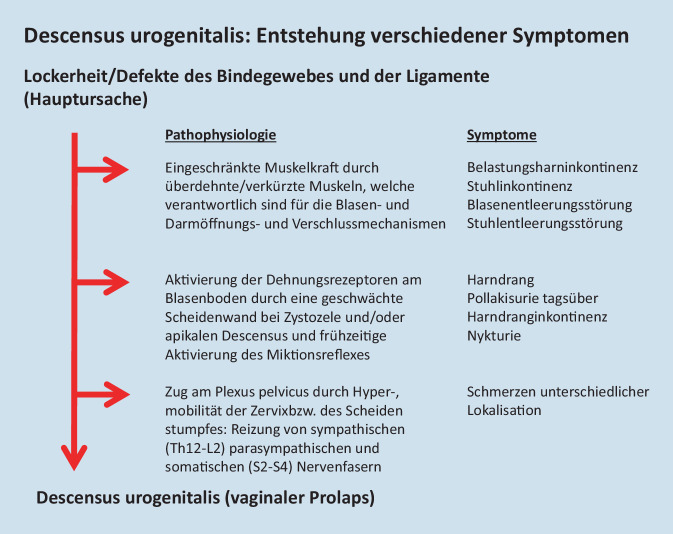
Pathophysiologische Pfade der Entwicklung eines vaginalen Prolaps und von unterschiedlichen Symptomen

Die Abb. 3 zeigt in einer Übersicht die pathophysiologischen Mechanismen, die zu Belastungsharninkontinenz und Stuhlinkontinenz (Verminderung der Verschlusskräfte) sowie zu Blasenentleerungsstörung (obstruktive Miktion) und Stuhlentleerungsstörung (obstruktive Defäkation) durch Minderung der Öffnungskräfte führen.

Wenn die bindegewebigen Lockerungen zur Zystozele bzw. zur apikalen Senkung führen, kann der Blasenboden instabil werden, d. h. schon bei geringer Füllung können die Dehnungsrezeptoren frühzeitig gereizt werden, was zu Harndrangbeschwerden bis hin zu Pollakisurie, Harndranginkontinenz und Nykturie führen kann (Abb. 2; [13–15]).

Lange Zeit wurde nicht erkannt, dass urogenitaler Deszensus zu – auch massiven – Schmerzen führen kann. Mittlerweile sind allerdings die Mechanismen der Schmerzentstehung bei vaginalem Prolaps identifiziert [7, 16, 17]. Das Becken ist versorgt mit einem breit vernetzten Nervenplexus, dem Plexus pelvicus, der sympathische Fasern (von Brustwirbelkörper [BWK] 12–L2 über postganglionäre hypogastrische Fasern), parasympathische Fasern (über S2–S4), somatische Nerven (von S2–S4) und das Ganglion Frankenhäuser enthält. Im Falle eines urogenitalen Deszensus bewirkt die Hypermobilität der Zervix bzw. des Scheidenstumpfes eine Dehnung von Nervenfasern dieses Nervenplexus, was zu Hypersensitivität und Schmerzen führen kann. Die Symptomatik kann viszeralen Charakter haben, es können Schmerzen im Unterbauch und im tiefen Kreuz auftreten, die auch in die Vagina und den Scheidenausgang ausstrahlen können [18].

Neuroinflammatorische Effekte inklusive Mastzellaktionen [19] können ebenfalls eine Bedeutung in der Schmerzentstehung beim urogenitalen Deszensus haben. Diese sind ggf. auch in der Auslösung einer interstitiellen Zystitis beteiligt, die häufig mit Schmerzen verbunden ist („bladder pain syndrome“, BPS; [20]). Da die interstitielle Zystitis/BPS [21] bei Frauen ca. 9-mal häufiger auftritt als bei Männern, ist ein weiblicher Faktor in der Entstehung (möglicherweise ein urogenitaler Deszensus?) zu vermuten.

Bei der Betrachtung der einzelnen Symptome bei Frauen mit vaginalem Prolaps muss berücksichtigt werden, dass die Assoziationen hier überwiegend nicht-linear sind [22, 23]. So korreliert der Widerstand des Harnflusses in der Urethra indirekt in der 4. Potenz zum Harnröhrendurchmesser (Hagen-Poiseuille-Gesetz). Gleichzeitige Minderungen der Öffnungs- und Verschlusskräfte können Symptome verändern und gegenläufige Effekte erzielen (z. B. okkulte Belastungsharninkontinenz). So gibt es durchaus Frauen, die mit höhergradigem vaginalem Deszensus kaum Symptome entwickeln.

## Ergebnisse chirurgischer Symptomheilung durch adäquate Prolapskorrektur

Da die genannten Symptome in den meisten publizierten Studien prä- und postoperativ nicht beschrieben wurden, besteht großer Bedarf an neuen Studien, um die Lebensqualität bei Frauen mit stark belastenden Symptomen zu verbessern.

Die PROpel-Studie (registriert unter ClinicalTrials.gov-Identifier: NCT00638235) beobachtete viele Symptome („patient-related outcome measures“, PROM) präoperativ und bis zu 2 Jahren postoperativ unter Verwendung eines validierten Fragebogens, so dass die Ergebnisse hier veranschaulicht werden können.

## Patienten und Methoden

In der PROpel-Studie unterzogen sich 277 Frauen mit symptomatischem vaginalem Prolaps der POP-Q-Stadien II–IV (Pelvic Organ Prolapse-Quantification) einer Prolapskorrektur mit „Elevate anterior/apical“ oder „Elevate posterior/apical“. Alle Frauen beantworteten 46 Fragen des Pelvic Floor Disorder Questionnaires präoperativ, 6, 12 und 24 Monate postoperativ und wurden bei Kontrolluntersuchungen POP-Q-Messungen unterzogen.

Hierbei wurde die Häufigkeit von Symptomen mäßiger oder starker Ausprägung im Gegensatz zu den übrigen Ausprägungen als relevant betrachtet: keine Beschwerden, Beschwerden ja, aber ohne Leidensdruck, geringe Beschwerden. Um den Einfluss einer guten und weniger guten operativen Rekonstruktion abzuschätzen, wurden diejenigen Frauen als anatomische Responder betrachtet, die zu jedem postoperativen Zeitpunkt (6, 12 und 24 Monate) und in jedem Kompartiment (anterior, apikal und posterior) das POP-Q-Stadium 0 oder I erreichten. Die übrigen Frauen wurden als anatomische Non-Responder gesehen. Diese entwickelten in bis zu 63 % Zystozelen 2. Grades und in 27 % Rektozelen 2. Grades innerhalb von 2 Jahren, während sie apikal in 84 % das Stadium 0 oder 1 erreichten (s. auch Tabelle in [25]).

## Ergebnisse bei Blasenentleerungsstörungen

Aus Tab. 2 geht hervor, dass präoperativ in ca. einem Drittel des untersuchten Kollektivs das Symptom einer Blasenentleerungsstörung mäßiger und starker Ausprägung berichtet wurde. Frauen mit zweitgradigem Prolaps in der POP-Q-Messung wiesen ähnlich häufig Symptome auf wie diejenigen mit POP-Q-Stadium III–IV. Frauen mit Rektozelen wiesen eine etwas niedrigere (nicht-signifikante) Häufigkeit auf (s. auch [25] mit kompletter Darstellung der Daten). Die Frauen mit POP-Q-Stadium II hatten mit 69 % ähnlich hohe Heilungsraten wie diejenigen mit POP-Q-Stadien III–IV. Die posteriore/apikale Rekonstruktion bei Rektozelen brachte Heilungsraten von 64 %, die anteriore/apikale Rekonstruktion bei Zystozelen 76 %.

**Tab. 2:** Häufigkeiten und Heilungsraten des Symptoms schwacher Harnstrahl/verlängerte Miktionszeit in Abhängigkeit vom POP-Q-Stadium (Pelvic Organ Prolapse-Quantification), bei Rekto- und Zystozelen sowie in Abhängigkeit vom anatomischen Erfolg der Prolapskorrektur. (Mod. nach [25])

Schwacher Harnstrahl/verlängerte Miktionszeit					
Häufigkeit mäßiger oder starker Beschwerden					
Teilkollektiv	Präoperativ	2 Jahre postoperativ	P	RR prä-/postoperativ	Heilungsrate (Verschwinden mäßiger und starker Beschwerden) (%)
POP-Q-Stadium II	35,2 % (*n* = 122)	11 % (*n* = 91)	< 0,01	3,2	69
POP-Q-Stadium III–IV	33,3 % (*n* = 150)	9 % (*n* = 89)	< 0,01	3,7	73
*p*	0,74	0,66	–	–	–
Posterior-apikale Rekonstruktion bei Rektozelen	28,1 % (*n* = 135)	10,0 % (*n* = 110)	< 0,01	2,8	64
Anterior-apikale Rekonstruktion bei Zystozelen	38,7 % (*n* = 142)	9,3 % (*n* = 75)	< 0,01	4,2	76
*p*	0,10	0,80	–	–	–
Responder	31,2 % (*n* = 141)	4,2 % (*n* = 95)	< 0,01	7,4	87
Non-Responder	39,1 % (*n* = 87)	19,7 % (*n* = 61)	< 0,01	2,0	50
*p*	0,12	< 0,01	–	–	–

Interessant ist der Vergleich der Responder vs. Non-Responder. Während bei den gut rekonstruierten Respondern Heilungsraten in 87 % erzielt wurden, war die Heilungsrate bei den Non-Respondern mit 50 % signifikant geringer.

## Ergebnisse bei Harndranginkontinenz und Nykturie

Symptome von Harndrang, Pollakisurie tagsüber, Harndranginkontinenz und Nykturie traten bei diesem Frauenkollektiv in 39–48 % präoperativ auf. Die kompletten Datensätze sind in den entsprechenden Publikationen einsehbar [26, 27]. Hier werden wie bereits zuvor die Auswirkungen einer guten anatomischen, langfristigen Prolapskorrektur (Frauen, die zu jedem Nachuntersuchungszeitpunkt und in jedem Kompartiment das POP-Q-Stadium 0 oder I erreichten, werden als Responder bezeichnet) den übrigen Frauen mit schlechterem anatomischem Ergebnis (Non-Responder genannt) gegenübergestellt (Tab. 3). Während bei den Respondern Heilungsraten von 82 % (Harndranginkontinenz) und 92 % (Nykturie) zu beobachten waren, betrugen die Heilungsraten bei den Non-Respondern nur 46 und 39 % (Unterschiede zu Respondern signifikant).

**Tab. 3:** Häufigkeit und Heilungsraten der Symptome Harndranginkontinenz und Nykturie mäßiger und starker Ausprägung in Abhängigkeit vom POP-Q-Stadium (Pelvic Organ Prolapse-Quantification), bei Rekto- und Zystozelen sowie in Abhängigkeit vom anatomischen Erfolg der Prolapskorrektur (Responder vs. Non-Responder). (Mod. nach [27] und [26])

	Häufigkeit präoperativ	Häufigkeit 2 Jahre postoperativ	*p*	RR	Heilung (Verschwinden mäßiger und starker Beschwerden) (%)
*Harndranginkontinenzsymptome mäßiger oder starker Ausprägung*					
Responder (*n* = 141)	39,0 %	7,4 %	< 0,01	5,3	82
Non-Responder (*n* = 87)	40,2 %	21,7 %	< 0,01	1,9	46
*p*	0,85	< 0,01	–		
*Nykturiesymptome mäßiger oder starker Ausprägung*					
Responder (*n* = 141)	39,0 %	3,2 %	< 0,01	12,2	92
Non-Responder (*n* = 87)	48,3 %	29,5 %	< 0,01	1,6	39
*p*	0,67	0,019	–		

## Ergebnisse bei Schmerzen vor und nach Prolapskorrektur

In der PROpel-Studie wurden 6 Schmerzsymptome beobachtet, die in anteriore, viszerale und posteriore Schmerzen gruppiert wurden [28] und präoperativ in 24–40 % auftraten (Tab. 4).

In der PROpel-Studie wurden 6 Schmerzsymptome beobachtet

Ein Schmerzsymptom mäßiger oder starker Ausprägung hatten präoperativ 67 % der Frauen im Kollektiv. Alle Schmerzsymptome besserten sich hochsignifikant, so dass Heilungsraten 2 Jahre postoperativ von 53 % bzw. 58 % (posteriore Lokalisation), 88 % bzw. 90 % (viszerale Beschwerden) und 83 bzw. 88 % bei anterioren Schmerzen beschrieben wurden.

**Tab. 4:** Häufigkeit von Schmerz unterschiedlicher Lokalisation mäßiger oder starker Ausprägung (R2) präoperativ und 24 Monate postoperativ mit Angabe der Heilungsraten, wobei Heilung definiert ist mit Verschwinden mäßiger und starker Beschwerden

PFDI-Fragen zu Schmerzen	Schmerzlokalisation	Häufigkeit von R2 präoperativ (*n* = 277) (%)	Häufigkeit von R2 2 Jahre postoperativ (*n* = 185) (%)	*p*	RR prä-/postoperativ	Heilungsrate (Verschwinden mäßiger und starker Beschwerden) (%)
Druck unteres Abdomen	Anterior	32,9	5,4	< 0,01	6,1	83
Schmerz unteres Abdomen oder Genitalregion		22,8	2,7	< 0,01	8,4	88
Schweregefühl und Dumpfheit im Beckenbereich	Viszeral	27,2	2,7	< 0,01	10,1	90
Unwohlsein im Becken im Stehen, bei körperlicher Aktiv		40,8	4,9	< 0,01	8,3	88
Schmerzen unteres Kreuz	Posterior	37,9	17,8	< 0,01	2,1	53
Bauch- oder Kreuzschmerzen bei Belastung		24,6	10,3	< 0,01	2,4	58

*p* < 0,01 (mod. nach [28])

*PFDI* „pelvic floor disorder inventory“

## Ergebnisse bei anorektalen Dysfunktionen prä- und postoperativ

In der Studie wurden auch mehrere Symptome anorektaler Dysfunktionen präoperativ und bis 2 Jahre postoperativ abgefragt [29]. Überraschend war, dass nicht nur die Symptome der obstruktiven Defäkation mit 71–84 % geheilt wurden, sondern auch die Symptome der Stuhlinkontinenz unterschiedlicher Schweregrade in 58–72 %. Auch die selteneren Hämorrhoidalbeschwerden und Beschwerden eines Rektumprolapses wurden signifikant besser (Tab. 5).

**Tab. 5:** Heilungsraten anorektaler Dysfunktionen 2 Jahre nach vaginaler Prolapskorrektur mit Elevate. (Mod. nach [29])

PFDI-Fragen zu anorektalen Dysfunktionen	Prä- und postoperative Häufigkeiten und Heilungsraten von Symptomen mäßiger oder starker Ausprägung				
	Häufigkeit präoperativ (*n* = 277) (%)	Häufigkeit 2 Jahre postoperativ (*n* = 185) (%)	*p*	RR (prä-/postoperativ)	Heilung (Verschwinden mäßiger und starker Beschwerden) (%)
*Stuhlinkontinenz*					
1. Grades	33,2	14,0	< 0,001	2,37	58
2. Grades	17,7	4,9	< 0,001	3,61	72
3. Grades	6,1	2,1	< 0,05	2,90	66
*Obstruktive Defäkation*					
Muss auf Vagina oder um das Rektum drücken für Stuhlgang	28,2	6,0	< 0,001	4,70	79
Gefühl stark drücken zu müssen, um Stuhlgang zu haben	32,9	5,4	< 0,001	6,09	84
Gefühl der unvollständigen Stuhlentleerung	29,6	8,6	< 0,001	3,44	71
*Sonstige Stuhlbeschwerden*					
Hämorrhoidalbeschwerden	21,0	10,8	< 0,01	1,94	49
Rektumprolapsbeschwerden	7,9	3,2	< 0,05	2,47	59

*PFDI* „pelvic floor disorder inventory“

## Diskussion und Schlussfolgerung

In den letzten 30 Jahren wurden neue Grundlagen für das Verständnis der Beckenbodenfunktionen und der Beckenbodendysfunktionen erarbeitet [5, 6, 8, 16]. Hierbei wurden neue Vorstellungen der funktionellen Anatomie und auch der Pathophysiologie von Belastungsharninkontinenz, Harndrangbeschwerden, Nykturie, Harnblasenentleerungsstörung, Schmerzen und anorektalen Dysfunktionen erarbeitet, die durch urogenitalen Deszensus verursacht werden können. Während sich für die Belastungsharninkontinenz sehr effektive Methoden herausgebildet haben und diese auch angewandt werden [30–32], wird für Harndrangbeschwerden und Stuhlinkontinenz bei Frauen mit vaginalem Prolaps nur von wenigen Zentren eine chirurgische Prolapskorrektur zur Symptomheilung in Erwägung gezogen [33].

Erstmals wurden in der registrierten Multicenter-PROpel-Studie sehr ausführlich mit validierten Fragebögen viele Symptome präoperativ und im Verlauf bis zu 2 Jahren postoperativ beobachtet und analysiert. Es konnte gezeigt werden, dass viele dieser Symptome hochsignifikant gebessert werden konnten und hohe Heilungsraten erzielt wurden.

Der Vergleich anatomischer Responder vs. Non-Responder hat gezeigt, dass auch der operative Erfolg der anatomischen Korrektur signifikanten Einfluss auf die Heilungsraten hatte. So konnte in diesem selektierten Krankengut die Harndranginkontinenz in bis zu 82 %, die Nykturie sogar in bis zu 92 % und Blasenentleerungsstörung in bis zu 87 % nach langfristig guter Prolapskorrektur bei den Respondern geheilt werden. Wesentlich dürfte eine optimale Fokussierung auf eine achsengerechte, ligamentäre Rekonstruktion sein. So haben unterschiedliche Autoren damit ähnlich hohe Heilungsraten erzielen können [33]. In einer Literaturanalyse haben DeBoer et al. [34] zwar eine starke Assoziation zwischen vaginalem Prolaps und Symptomen überaktiver Blase gefunden, bei Berücksichtigung herkömmlicher Techniken zur Prolapskorrektur allerdings keine überzeugenden Ergebnisse der RR (relative Ratios präoperativ/postoperativ) sehen können.

Der operative Erfolg der anatomischen Korrektur hat signifikanten Einfluss auf die Heilungsraten

Nach laparoskopischer Promontofixation – die nicht einer achsengerechten Rekonstruktion entspricht – wurde eine De-novo-Stuhlinkontinenz von 5 % beobachtet, während sich die Symptome der Harndranginkontinenz postoperativ vs. präoperativ nicht veränderten [35].

Die laparoskopische Verwendung autogener Sehnen sollen netzassoziierte Schmerzen reduzieren, allerdings erfolgte in dieser aktuellen Arbeit keine ausreichende Symptombeobachtung, so dass deren Bewertung schwierig ist [36].

Insgesamt kann als Schlussfolgerung festgestellt werden, dass eine achsengerechte anatomische Rekonstruktion dazu führen kann, dass entsprechend der pathophysiologischen Vorstellungen (Optimierung der Ansatzareale der Beckenbinnenmuskulatur zur Verbesserung der Öffnungs- und Verschlusskräfte) Belastungsharninkontinenz, Stuhlinkontinenz, aber auch Miktion und Stuhlentleerung gebessert bzw. geheilt werden können. Mit der Erzielung einer stabilen vorderen Scheidenwand (zur Unterstützung der Blase) und eines stabilen Apex der Vagina lassen sich sowohl Symptome wie überaktive Blase als auch Schmerzen bei Frauen mit urogenitalem Deszensus wesentlich bessern. Die Erkenntnis, dass sogar eine Rektozelenkorrektur durch posteriore/apikale Rekonstruktion die Blasenentleerungsstörung beheben kann, weist ebenfalls auf die Bedeutung der Muskelkräfte zur Öffnung des Blasenhalses bei Miktion hin, wie bereits in anderen Studien festgestellt [33, 37].

In diesem Zusammenhang soll festgestellt werden, dass (zumindest in niedrigen Prolapsstadien) Physiotherapie signifikante Symptomverbesserung erbringen kann [38] und konservative Therapien somit in der Regel vorgeschaltet werden [39].

Physiotherapie kann in niedrigen Prolapsstadien signifikante Symptomverbesserungen erbringen

Es wird Aufgabe des neuen Arbeitskreises „Rekonstruktive Beckenbodenchirurgie“ der Deutschen Gesellschaft für Urologie e. V. sein, diese neuen Erkenntnisse zu vermitteln. Es soll ein Wandel hin zu einer Beckenbodenchirurgie eingeleitet werden, die das Ziel der Symptomheilung sowohl von Blasenfunktionsstörungen, Schmerzen als auch anorektaler Dysfunktionen hat und damit eine Verbesserung der Lebensqualität erzielen kann. In systematischen Literaturanalysen und auch durch Durchführung neuer Studien werden die aktuell unterschiedlich eingesetzten operativen Techniken zur Prolapskorrektur hinsichtlich dieses Ziels zu überprüfen sein.

## Fazit für die Praxis

- Beim urogenitalen Deszensus mit den entsprechenden Symptomen handelt es sich um eine Volkskrankheit, die die Lebensqualität stark beeinträchtigt und große Kosten verursacht.
- Wesentlich für die Entwicklung eines vaginalen Deszensus sind bindegewebige Lockerungen bzw. Defekte.
- Während bei den gut rekonstruierten Respondern Heilungsraten in 87 % erzielt wurden, war die Heilungsrate bei den Non-Respondern mit 50 % signifikant geringer. Wesentlich dürfte eine optimale Fokussierung auf eine achsengerechte, ligamentäre Rekonstruktion sein.
- Für die Belastungsharninkontinenz und für symptomatische Prolapszustände haben sich sehr effektive Methoden herausgebildet.
- Langfristige Verbesserung/Heilung von Schmerzen, Symptomen über-,  unteraktiver Blase und anorektalen Dysfunktionen sind wichtige Ziele der Prolapskorrektur.
